# 

**Published:** 1935

**Authors:** 


### CONTENTS.

| | Page |
|---|---|
| The Evolution of British Orthopædics. By Sir Humphry Rolleston, Bart., G.C.V.O., K.C.B., M.D. .. .. .. | 143 |
| Diseases of the Lachrymal Sac. By A. E. Iles, O.B.E., M.B., F.R.C.S. .. .. .. .. .. .. .. .. .. | 151 |
| Treatment of Lachrymal Obstruction. By Gordon R. Scarff, M.B., Ch.B., F.R.C.S. .. .. .. .. .. .. .. | 159 |
| Thomas Marryat, M.D.: A Memoir. By A. S. MacNalty, M.D., F.R.C.P. .. .. .. .. .. .. .. .. | 165 |
| Reviews of Books .. .. .. .. .. .. .. .. | 171 |
| Editorial Notes .. .. .. .. .. .. .. .. | 181 |
| Meetings of Societies .. .. .. .. .. .. .. | 182 |
| The Medical Library of the University of Bristol .. .. | 186 |
| Publications Received .. .. .. .. .. .. .. | 188 |
| Local Medical Notes .. .. .. .. .. .. .. | 189 |

